# Investigation of Strongyle Prevalence and Associated Risk Factors in Horses in and around Alage District, Ethiopia

**DOI:** 10.1155/2022/3935008

**Published:** 2022-08-12

**Authors:** Mulugeta Tesfaye Alemayehu, Belete Kuraz Abebe, Sharew Mekonnen Haile

**Affiliations:** Department of Animal Science, Werabe University, P.O. Box 46, Werabe, Ethiopia

## Abstract

**Background:**

Horses are used for a variety of purposes in Ethiopia. However, their service is hampered by a variety of health issues. Strongylosis is a parasitic infestation of the gastrointestinal tract that has a significant impact on the working ability, reproductive performance, well-being, and welfare of horses. The existence of Strongylosis in the study area is reported from clinical cases; however, its prevalence has not been well studied.

**Objective:**

The current study was carried out from January 2019 to July 2019, to determine the prevalence and associated risk factors of Strongyle in horses in and around Alage district.

**Methods:**

A cross-sectional study design was used, and 384 horses were sampled from three peasant associations inseparably. The floatation technique was used in laboratory analysis.

**Result:**

Strongylosis was discovered in 67.19 percent of the cases. In Naka, Dilbato, and Koricho peasant associations, the infestation magnitudes were 64.1%, 68%, and 69.5%, respectively. The increased level of animal-related prevalence was observed in male (68.1 percent), young (84.4 percent), and poor body condition (90 percent) horses. Age and body condition scores were statistically significant associations with the disease under study at *p* ≤ 0.000. While sex and peasant associations did not predict the problem significantly (*p* ≤ 0.05), young horses and horses in poor and medium body conditions are 4.66 (CI: 2.22–9.76), 9.63 (CI: 3.77–24.63), and 1.93 (1.03–3.60) times more likely to be infected with Strongylosis, respectively.

**Conclusions:**

The occurrence of Strongylosis is determined independently by age and BCS. Strongyle infestations are common in horses in the study area, posing a significant barrier to horse production and well-being. As a result, appropriate disease prevention and control measures should be implemented.

## 1. Introduction

Ethiopia hosts the largest equine population in Africa, with estimated number of 2.11 million horses, 8.98 million donkeys, and 0.38 million mules, widely distributed throughout the country [1]. From available developed and mature horses, about 1.35 million were used for carriage like riding, carting and packing, 0.25 million were for draught and plowing, and the rest 0.12 million were used for horse race, wedding ceremony, and religious activities, as well as for warfare purpose [1]. Horse and other equine force is crucial for transportation where the road and other infrastructures are scarce; in such places the most basic necessities, village supplies, and other logistics are transported by the aid of equine forces [2].

Even if equines are used for many tasks in Ethiopia, their service is impaired by a number of health problems [3]. Among these, the most common agents leading in to illness, suffering, and fatality are infectious diseases and parasitism (endo-parasites), which resulted in considerably reduced animal working ability and reproductive performance and also reach up to loss of their life [4]. Endoparasites are sustained to be a substantial hazard to the welfare of equines. In both extensive and intensive farming systems, as the age of animal increases, the calculated samples for the current study were 384 heads of horses (Equation (1)). From the sampled animals, female constitutes 307 heads while male covers 77 heads [5].

Among these, Strongyles are gastrointestinal parasites belonging to the superfamily Strongyloidea, family Strongylidae, and genus Strongyle, and the genus comprises three species: *S. vulgaris*, *S. edentatus*, and *S. equines*. These parasites are abundant, and the adults are commonly found in the large intestine of affected horse for the sake of shelter and food [6]. From the Strongyle species, *Strongylus vulgaris* is the most economical, where its prevalence approaches to 100% in youngster's [7]. These parasites are vital because they migrate in the blood circulation and vital organs and can cause severe damage which might be life threating [8].

Of the predisposing factors, age is the most predictive to Strongyle infestation because young and undernourished horses have no well-developed immunity compared to the others. Once the animal becomes older, their disease resistance ability will also increase; as such moment, the parasite does not harm the animal strongly like it did to young foals [9]. The amount of infective larvae ingested by the new host and the age of the horse determine the severity of the disease and the clinical sign developed by host [10]. The most clinical signs observed during Strongylosis infestations are not specific to the problem, such as loss of condition, loss of muscle, irregular hair coat, debilitated immunity, high body temperature, colic, and loss of appetite [11]. “Verminous aneurysm” is a pathognomonic sign to Strongylosis [12]. Strongylosis is a severe health illness in foal reared in extensive pasture; it may also cause severe health problem in adult horses exposed to overcrowding and improper management [13].

In the study area, information on states of Strongylosis is not well established quantitatively as well as qualitatively. This may be due to lack of attention given to equine production sector. The absence of research activity in animal diseases, poor veterinary development, and lack of awareness to the economic impact of the disease has contributed to the less amount of information observed. However, the studies done in different district of the country are evident for the existence of Strongylosis infestation in horse, which has been demonstrated by different authors with varying prevalence. For instance, [14–19] revealed 87.97, 48.2, 53.74, 24.74, 59.25, and 53.3, respectively.

Even if the clinical cases reported from the veterinary offices suggest the existence of Strongylosis, to date, the occurrence of Strongylosis has not been well investigated in Alage and its surrounding so far. So, the present study therefore is aimed at investigating the prevalence and associated risk factors of Strongylosis, in Alage and its surrounding with the following specific objectives:

- To investigate the prevalence of Strongyle infestation
- To assess the potential risk factors of Strongyle infestation in horse

## 2. Materials and Methods

### 2.1. Description of the Study Area

This research was conducted in and around Alage Agricultural Technical and Vocational Educational and Training College, as well as the nearby kebeles (peasant associations). Naka, Dilbeto, and Koricho Peasant Associations (PA) are located in Jido Kombolcha and Alaba districts, south-east and south-west of Alage. Alage is 217 kilometers south-west of Addis Ababa, at longitude 38°30 East and altitude 7°30 North, with an elevation of 1600 meters above sea level.

### 2.2. Study Design

The study was conducted from January 2019 to July 2019 in Alage district, Ethiopia. A cross-sectional study design was used.

### 2.3. Study Population and Their Management

The study populations were horses of all age groups; different body conditions, both sexes, and all physiological states were included.

### 2.4. Sampling and Sample Size Determination

Simple random sampling technique was used to select individual study animals. All animals included in this study were local breeds and reared under extensive production system. The sample size for this study was determined as described by [20] as follows.

(1) $$\begin{array}{c} \frac{N=1.96\text{Pexp }{\left(1-\text{Pexp}\right)}^{2}}{d^{2}}, \end{array}$$

where *n* is the required sample size, Pexp ≤ expected prevalence, and *d* is the desired absolute precision.

In the study area, there were no previous reports of prevalence of Strongylosis. Therefore, the average expected prevalence rate was assumed to be 50% for the area within 95% confidence

interval (CI) at 5% desired precision as stated by [20]. Hence, using the formula, the calculated samples for the current study were 384 heads of horse of both sexes of all age groups (Equation (1)). Sampled animal females were constitutes 307 heads while male covers 77 heads.

### 2.5. Sampling Collection

The fecal samples were collected directly from the rectum of the horse by wearing disposal glove and transferred in to air tight screw cupped labeled container and transported to Alage Veterinary Parasitology laboratory immediately after collection. During sample, all necessary information was recorded.

### 2.6. Carpological Examination

The floatation technique [11] was conducted to concentrate parasite eggs from feces and examined under a microscope (10x and 40x) for the presence of parasite egg. The eggs were identified based on their morphology [21]. The ages were determined by looking the teeth of horse [22].

### 2.7. Data Analysis

The data obtained were classified, filtered, and coded into Microsoft Excel® 2010. The data was exported to SPSS version 20 for appropriate statistical analysis. The results were presented in descriptive statistics by frequency and percentage. Analytical statistics, chi-square (*χ*^2^), and odds ratio (OR) were employed for the purpose of identifying the risk factors associated with disease of interest.

### 2.8. Ethical Consideration

Before any attempt to collect sample, the protocols were approved by Alage ATVET, College Animal Research Ethical Committee. Official permission was obtained from the Agricultural Administration Office of the Zone and Districts.

## 3. Result

### 3.1. Descriptive Analysis of Strongylosis

In the present study, 384 fecal samples were examined in laboratory by using flotation method. Accordingly, out of 384 horses sampled, 258 contain Strongyle egg. The overall prevalence of Strongyle in study area was 67.19%. The bivariate analysis computed indicates the presence of statistically significant differences between positive and negative test results at *p* ≤ 0.000. This also indicates that Stronglosis is strongly prevalent and becomes a big problem in the study area (Table 1).

### 3.2. Association of Strongylosis with Its Risk Factors

According to the present study, Strongyle infestation was highly prevalent in the horses in the study area. From 254 horses positive for the disease under investigation, the prevalence rate was higher in Koricho (69.5%) followed by Dilbato (68%) and Naka (64.1%) PA. From 307 stallions sampled, 209 (68.1%) were positive for Strongyle egg while 63.6% of mares show positive for the problem of interest. Similarly, the infestation rate of Strongylosis was higher in horses have poor body condition score 90% than medium body condition score 63%, while the horses with good body condition score were constitute 52.6% and also young horses were more prevalent (84.4%) to Strongyle infestation followed by adult 58.6% and old 51.9% horses. The chi-square test was computed to observe association between the disease of interest and potential risk factors. Hence, only age and body condition scores show statistically significant association with Strongyle at *χ*^2^ = 30.8, *p* ≤ 0.000 and *χ*^2^ = 26.18, *p* ≤ 0.000, respectively, whereas peasant association and sex had no statistically significant association with the problem of interest at *p* ≥ 0.05 (Table 2).

### 3.3. Risk Linked Potential Risk Factor Analysis of Strongylosis

The animal level Strongylosis and their association with exposure variables were computed using logistic regression. Accordingly, the prevalence of Strongylosis did not show significant variations among Pa and sex (*p* ≥ 0.05) using univariate logistic regression analysis. However, age and body condition scores were the two potential risk factors significantly associated with Strongylosis (*p* ≤ 0.05) according to univariable analysis of logistic regression. Young and adult animals were 4.66 (95% CI: 2.22-9.76 and *p* ≤ 0.000) and 1.177 (95% CI: 0.62-2.24 and *p* ≤ 0.63) times at higher risk of being infected with Strongylosis than old horses, while animals that had poor body condition score were 9.63 times at higher risk of being positive to Strongyle infestation, and also horses had medium body condition score, were 1.93 times at higher risk than animals had good body condition score (Table 3).

### 3.4. Risk Linked Predictors of Strongylosis

A risk linked body condition score and age were observed in the final model of animal level analysis. Results of multivariable analysis indicated that an advance in age and amassed body condition score of horse were significantly associated with decreased odds of higher intensity Strongylosis. The odds of young horse not infected by Strongylosis were decreased by 0.112 (95% CI: 0.044–0.284; *p* ≤ 0.000), the probability of adult horse being infected by Strongylosis was increased by 53.6% (0.56; 95% CI: 0.288–0.957; *p* ≤ 0.049), while the odds of poor body conditioned horse infected by Strongylosis were decreased by 0.209 (95% CI: 0.100–0.437, *p* ≤ 0.000). Thus, in the multivariable analysis, both body condition score and age were remained to be independently associated with Strongylosis whereas others not. However, only young, adult, and poor body conditioned horses significantly predicted the problem of interest at *p* ≤ 0.000, *p* ≤ 0.049, and *p* ≤ 0.000, respectively (Table 4).

## 4. Discussion

Horses are exposed to helminth parasites from various groups all over the world, posing a significant threat to their health and causing irreversible internal damage, morbidity, and mortality [15]. Strongyle nematodes are a common equine gastrointestinal parasite, with adults commonly parasitizing horses' large intestines [6]. In the current study, the sample was collected from three points of nearly different climatic-conditioned peasant associations; these kebeles showed nearly equal presence of infestation, and no statistically significant difference in degrees of infestation was observed. This supported the notion that, because the Strongyle is so common in nature, horses in the study area could become infected with no discernible difference. In the current study, 258 horses out of 384 tested positive for Strongyles, resulting in a 67.19 percent overall prevalence in horses. This suggests that these parasites are extremely common and widespread in the study area. This finding is consistent with other researchers [19]. In contrast to the current findings, researchers have reported a higher prevalence of Strongyles from various parts of the continent [23, 24] and lower prevalence also reported from other findings [25]. The variation in prevalence is caused by differences in the study area, the season of the study, and deworming strategy differences. The prevalence of Strongyle was found to be statistically significant at *p* ≤ 0.0001 in the current study, which is consistent with the findings of [14] who discovered a statistically significant association between the host and the parasite understudy at *p* ≤ 0.05. The host plays an important role in disease susceptibility, with the horse being the most vulnerable to infestation, and the disease is extremely severe in weakened, debilitated, or immune-compromised horses [26].

Due to the ubiquitous nature of Strongylosis, horses of all age groups are affected, with various degrees of prevalence rate in accordance to the age-wise analysis. In the current study, the infestation rate of Strongyle revealed that younger horses appeared to be more susceptible to the disease under study. The findings are consistent with those of [27–29] Researchers, on the other hand, investigated and reported that adult and older horses harbored more Strongylosis than young horses, as recognized by [15], in adults, while [30] recognize that in older horses. Both arguments run counter to the current outcome. However, in the current study, younger age groups showed a high degree of infestation and a statistically significant difference was observed at *p* ≤ 0.000. This suggests that age is one of the risk factors for the occurrence of Strongyle infestation in horses. Statistically, significant differences were also reported by [15, 24].

In contrast to our findings, [19, 30] found statistically insignificant differences between age groups at *p* ≤ 0.05. The current study's findings support previous researchers' findings, which can be explained by the fact that younger animals may be more susceptible to Strongyle infestation than older animals. Because infestations in horses are more visible in young and undernourished horses, and the aged horses appear to gradually develop immunity against the GI parasites and are not severely affected by parasite-related problems; age is an important risk factor for susceptibility to Strongyle infestation [9].

Sex is one of the intrinsic factors expected to cause Strongylosis, and in the current study, male horses appear to be more susceptible to the infestation than female horses. There was no statistically significant difference in Strongyle infestation rates based on gender (*p* ≤ 0.27). The current study's findings are supported by the work of [18] who indicate that stallions were more prone to the disease of interest, despite having a negligible association with the problem. Female horses were found to be more infected with Strongylosis by [28]. However, there is no statistically significant relationship between parasitic intensity and sex. The agreement of these studies could be due to females' not being exposed to new areas and being kept in similar pastures. While male horses are used for transportation of goods and other resources, they will travel long distances across different agroecological areas and will meet animals from different areas, which may increase the possibility of males becoming infected with Strongyle. Because of the common grazing system, sharing of shelter, similar deworming strategy, and lack of rotation in grazing due to land scarcity and other husbandry practices, there are no significant differences between parasite and horse gender. However, [31] discovered statistically significant differences between parasitic infestation and horse gender.

The body condition score was found to be a risk factor for the occurrence of Strongylosis in the current study area, with the magnitude of parasitism being greater in poor body conditioned horses than in others. Many other researchers agree with this finding, stating that Strongylosis is more common in horses with poor body condition [32, 33]. In contrast to our finding, [34] revealed a high degree of infestation in good body condition horses, while [18] noted maximum prevalence in medium body condition horses. The current study has also indicated a statistically significant difference (*p* ≤ 0.000) between body condition scores and Strongyle infestation. The same aspects of the current study were reported by [14], who found a significant relationship between body condition and parasite infestation in their respective study areas. This significant relationship indicates an increasing trend of Strongylosis in horses with poor body condition, which can be explained by the fact that animals with good body condition scores may be able to respond more easily to the pathogen under study than poorly conditioned horses. Malnourished animals have debilitated immunity as a result, making them easily vulnerable to the parasite, and even a small number of infective larvae will threaten the wellbeing of horses [14]. Even though Strongylosis is predicted by body condition score in the current study, some other findings are reported as statistically insignificant (*p* ≤ 0.05) by various researchers [33, 34]. The season of the study, the number of horses sampled, the types of production, and management differences could all contribute for the observed variations.

A bivariate analysis was performed to determine the impact of each factor on the occurrence of Strongylosis. According to the results of the analysis, age, and body condition score played a role in the occurrence of the infestation. As a result, young and adult horses are more likely to contract the infestation than older horses. Young horses are five times more likely than others to be infected with the Strongyle parasite and only young horses and were significantly associated with the disease of interest at *p* ≤ 0.0001. This finding is strongly supported by the work of other researchers such as [17]. In contrast to our findings, [16] found that age is not a predictor for the occurrence of Strongylosis at *p* ≤ 0.100 and *p* ≤ 0.843, respectively. Similarly, body condition score was a risk factor of the disease incidence in the study. Horses with a low body condition score are ten times more likely to have a Strongyle infestation, while horses with a medium body condition score are only two times more likely. Furthermore, both poor and medium-conditioned horses have been found to be statistically significant predictors of the disease of interest. The findings were consistent with those of [6]. The findings revealed that a horse's nutritional status and age are prerequisites for the development of solid immunity to withstand the severity of the infestation. Peasant association and horse sex, on the other hand, have no part in the occurrence of Strongylosis. In agreement, [13] reported similar cases.

## 5. Conclusion and Recommendation

The goal of this study is to determine the prevalence of Strongyle in horses and the risk factors associated with the problem under investigation, in and around the Alage district. Strongyle infestations are common in horses in the study area, posing a significant barrier to horse production and well-being. The infestation was the most severe in males, young, and poorly conditioned horses, as well as at the Koricho peasant association. Age and body condition scores were prime determinant for the occurrence of a disease in the study area, whereas sex and PA did not have a statistically significant contribution to the occurrence of the infestation. Furthermore, because of their immature and debilitated immunity, young and poorly conditioned horses are more likely to contract a Strongyle infestation. Therefore, in order to combat the rate of infestation in the study area, appropriate disease prevention and control measures, as well as raising awareness about the importance of horse feeding and regular deworming, should be implemented, as should additional epidemiological studies undertake.

## Figures and Tables

**Table 1:** Descriptive analysis of Strongylosis.

Factor	No. of examined horses	Prevalence (%)	Wald	OR	*p* value
Horse	384	67.2	43.482	2.048	0.000

OR: odds ratio.

**Table 2:** Association analysis of Strongylosis.

Factor	Number of Animal examined	Positive animal	Prevalence	*χ* ^2^ value	*p* value
Kebele					
Naka	128	82	64.1		
Dilbato	128	87	68	0.92	0.63
Koricho	128	89	69.5		
Sex					
Male	307	209	68.1	0.55	0.270
Female	77	49	63.6		
Age					
Young	141	119	84.4	30.8	0.000
Adult	191	112	58.6		
Old	52	27	51.9		
B. condition					
Poor	80	8	90		
Medium	258	91	63.2	26.18	0.000
Good	57	27	52.6		

*χ* ^2^: chi-square.

**Table 3:** Risk linked potential risk factors associated to Strongylosis.

Factor	No. of examined horses	No. of positive horses	Prevalence	OR	95% CI lower-upper	*p* value
Kebele						
Naka	128	82	64.1	0.65	0.36-1.15	0.138
Dilbato	128	87	68.0	0.91	0.52-1.62	0.758
Koricho	128	89	69.5	Ref.		
Sex						
Male	307	209	68.1	0.86	0.48-1.52	0.603
Female	77	49	63.6	Ref.		
Age						
Young	141	119	84.4	4.66	2.22-9.76	0.000
Adult	191	112	58.6	1.17	0.62-2.24	0.627
Old	52	27	51.9	Ref.		
Body condition						
Poor	80	8	90.0	9.63	3.77-24.63	0.000
Medium	258	91	63.2	1.93	1.03-3.60	0.039
Good	57	27	52.6	Ref.		

OR: odds ratio; CI: confidence interval; Ref.: reference.

**Table 4:** Multivariable analysis of potential risk factors associated to Strongylosis.

Factor	Test result		Bivariate			Multinomial logistic regression		
	Negative	Positive	OR	CI lower-upper	*p* value	OR	CI lower-upper	*p* value
Age								
Young	141	119	4.66	2.22–9.76	0.000	0.112	0.044–0.284	0.000
Adult	191	112	1.17	0.62–2.24	0.627	0.536	0.288–0.957	0.049
Old	52	27	Ref.	Ref.				
Body condition								
Poor	80	8	9.63	3.77–24.63	0.000	0.209	0.100–0.437	0.000
Medium	258	91	1.93	1.03–3.60	0.039	0.830	0.439–1.57	0.568
Good	57	27	Ref.	Ref.				

OR: odds ratio; CI: confidence interval; Ref.: reference.

## Data Availability

The data used for this research article are available from the corresponding author upon request.
